# Personalised screening tool for early detection of sarcopenia in stroke patients: a machine learning-based comparative study

**DOI:** 10.1007/s40520-025-02945-5

**Published:** 2025-02-20

**Authors:** Huan Yan, Juan Li, Yujie Li, Lihong Xian, Huan Tang, Xuejiao Zhao, Ting Lu

**Affiliations:** 1 School of Nursing, Zunyi Medical University, Zunyi, Guizhou China; 2 Department of Nursing, Guizhou Provincial People’s Hospital, Guiyang, Guizhou China; 3 School of Nursing, Guizhou University of Traditional Chinese Medicine, Guiyang, Guizhou China

**Keywords:** Stroke, Sarcopenia, Machine learning, Predictive model

## Abstract

**Background:**

Sarcopenia is a common complication in patients with stroke, adversely affecting recovery and increasing mortality risk. However, no standardised tool exists for its screening in this population. This study aims to identify factors influencing sarcopenia in patients with stroke, develop a risk prediction model and evaluate its predictive performance.

**Methods:**

Data from 794 patients with stroke were analysed to assess demographic and clinical characteristics. Variable selection was performed using least absolute shrinkage and selection operator (LASSO) regression, followed by multivariate regression analysis. Logistic regression (LR), random forest (RF) and XGBoost algorithms were used to construct prediction models, with the optimal model subjected to external validation. Internal validation was conducted via bootstrap resampling, and external validation involved an additional cohort of 159 patients with stroke. Model performance was assessed using the area under the curve (AUC), calibration curves and decision curve analysis (DCA).

**Results:**

Seven variables were identified through LASSO and multivariate regression analysis. The LR model achieved the highest AUC (0.805), outperforming the RF (0.796) and XGBoost (0.780) models. Additionally, the LR model exhibited superior accuracy, precision, recall, specificity and F1-score. External validation confirmed the LR model’s robustness, with an AUC of 0.816. Calibration and DCA curves demonstrated their accuracy and clinical applicability.

**Conclusions:**

A predictive model, presented as a nomogram and an online risk calculator, was developed to assess sarcopenia risk in patients with stroke. Early screening using this model may facilitate timely interventions and improve patient outcomes.

## Introduction

Stroke is the leading cause of adult disability, with approximately 80% of patients with stroke experiencing limb dysfunction [1]. Neurological damage and feeding difficulties following a stroke can lead to structural, metabolic and functional abnormalities in muscle tissues, ultimately resulting in muscle atrophy and structural changes that contribute to secondary sarcopenia, also referred to as stroke-related sarcopenia. This condition is characterised by reduced muscle mass and strength, along with somatic dysfunction [2, 3]. While sarcopenia affects around 15% of healthy older adults, its prevalence in patients with stroke can reach as high as 56% [4]. The early symptoms of sarcopenia are subtle and often go unnoticed, leading to a lack of timely attention. When coupled with stroke, sarcopenia exacerbates patient outcomes, increasing risks of infections, malnutrition and prolonged hospital stays [5].

Therefore, prevention through early and standardised screening is critical for managing sarcopenia and promoting recovery in patients with stroke [6]. Currently, screening for sarcopenia primarily relies on calf circumference measurements and the Strength, Assistance with walking, Rise from a chair, Climb stairs, and Falls (SARC-F) scale scores [7]. Although widely used among older adults, the relevance and accuracy of the SARC-F scale are limited in patients with stroke due to the complex conditions associated with neurologic damage [8]. Additionally, while skeletal muscle index assessment using computed tomography (CT) is a key diagnostic tool for sarcopenia, its application is constrained by high costs, radiation exposure and procedural complexity [9, 10]. These challenges make the clinical assessment of sarcopenia particularly difficult.

Existing predictive models for sarcopenia primarily target community-dwelling elderly populations, with limited research focused on patients with stroke. Traditional screening tools often rely on a single standard, overlooking individual patient characteristics. Integrating machine learning with easily accessible clinical data addresses these limitations by enabling more precise, personalised predictions. Such an approach can facilitate the early identification of high-risk patients and enhance preventive measures. This study aims to construct and evaluate three machine learning-based predictive models for sarcopenia in patients with stroke. The optimal model, selected through rigorous validation, is presented as a user-friendly tool to support personalised screening and risk assessment in clinical practice.

## Materials and methods

### Study design and population

This study was conducted in the rehabilitation, neurology and neurosurgery departments of a hospital and approved by the hospital's Ethics Committee (approval number: Ethical Approval Word (Research) [2023] No. 060). The training cohort consisted of 794 patients with stroke retrospectively enrolled between January 2021 and December 2023. For external validation, 195 patients were prospectively enrolled between January and May 2024. Inclusion criteria included: (1) age ≥ 18 years; (2) confirmed stroke diagnosis via CT or magnetic resonance imaging (MRI); and (3) absence of significant intellectual or cognitive dysfunction. Exclusion criteria were: (1) psychiatric disorders; (2) pre-stroke sarcopenia history (defined as recalled pre-stroke SARC-F score ≥ 4); (3) other neurological disorders; and (4) incomplete clinical data. Diagnostic criteria followed the 2019 guidelines of the Asian Working Group for Sarcopenia (AWGS) [11]. Sarcopenia was defined as reduced muscle mass (skeletal muscle index < 7.0 kg/m^2^ for men and < 5.7 kg/m^2^ for women), combined with decreased muscle strength (grip strength < 28 kg for men and < 18 kg for women) and/or impaired somatic function (5STS ≥ 12 s).

### Data collection

Clinical and demographic data were obtained from electronic medical records, including age, gender, smoking and drinking history, osteoporosis, stroke characteristics (number, location and type), limb dysfunction, diabetes, times since stroke, tube feeding, days of hospitalisation, Barthel Index (BI) score, Nutritional Risk Screening 2002 (NRS2002) score, body mass index (BMI), National Institutes of Health Stroke Scale (NIHSS) score, C-reactive protein, serum total cholesterol and serum albumin—comprising 20 variables in total. Two researchers independently collected data using a standardised survey and diagnostic criteria, cross-checking for consistency. Discrepancies were resolved by rechecking medical records or consulting medical professionals to ensure data accuracy and completeness.

### Statistical analysis

Statistical analysis was conducted using SPSS (version 25.0, IBM Corp., USA) and R (version 4.2.1, R Foundation for Statistical Computing, Austria). Categorical variables were presented as counts and percentages *n* (%) and analysed using the *χ*^2^ test. Continuous variables were tested for normality. Normally distributed data were presented as mean ± standard deviation and analysed using the independent samples *t* test, while non-normally distributed data were expressed as median and interquartile range (IQR) and analysed using the Mann–Whitney U test. Predictive variables were selected using least absolute shrinkage and selection operator (LASSO) regression (glmnet package) and further analysed using multivariate logistic regression. Prediction models were constructed using logistic regression (LR), random forest (RF) and XGBoost algorithms. Model performance was evaluated based on the area under the curve (AUC), accuracy, sensitivity, specificity and F1 score. Calibration and accuracy were assessed using the Hosmer–Lemeshow (HL) test and calibration curves, while clinical utility was evaluated through decision curve analysis (DCA). A *P* value < 0.05 was considered statistically significant.

## Results

### Demographic and clinical characteristics of patients

The demographic and clinical characteristics of the patients are summarised in Table 1. The prevalence of sarcopenia was found to be 37.53% (298 out of 794) in the training cohort and 38.36% (61 out of 159) in the validation cohort. No significant differences were observed in baseline characteristics between the two cohorts, except for age and diabetes (*P* > 0.05).

**Table 1:** Demographic and clinical characteristics of patients

Variable	Training cohort (*n* = 794)		Validation cohort (*n* = 159)		*P* value
	Sarcopenia (n = 298)	Non-Sarcopenia (n = 496)	Sarcopenia (n = 61)	Non-Sarcopenia (n = 98)	
Age (year)	69.76 ± 12.25	65.69 ± 11.21	68.59 ± 13.75	62.78 ± 10.80	0.032
Gender, n (%)					0.828
Male	163 (54.70)	224 (45.16)	49 (50.00)	30 (49.18)	
Female	135 (45.30)	272 (54.84)	49 (50.00)	31 (50.82)	
History of smoking, *n* (%)					0.168
No	151 (50.67)	261 (52.62)	56 (57.14)	36 (59.02)	
Yes	147 (49.33)	235 (47.38)	42 (42.86)	25 (40.98)	
History of drinking, n (%)					0.633
No	144 (48.32)	259 (52.22)	54 (55.10)	30 (49.18)	
Yes	154 (51.68)	237 (47.78)	44 (44.90)	31 (50.82)	
Osteoporosis, n (%)					0.654
No	140 (46.98)	250 (50.40)	50 (51.02)	25 (40.98)	
Yes	158 (53.02)	246 (49.60)	48 (48.98)	36 (59.02)	
Number of strokes, n (%)					0.481
1 time	85 (28.52)	134 (27.02)	27 (27.55)	13 (21.31)	
2 times	77 (25.84)	124 (25.00)	28 (28.57)	9 (14.75)	
3 times	56 (18.79)	123 (24.80)	22 (22.45)	23 (37.70)	
More than 3 times	80 (26.85)	115 (23.19)	21 (21.43)	16 (26.23)	
Limb dysfunction, n (%)					0.919
No	103 (34.56)	300 (60.48)	61 (62.24)	19 (31.15)	
Yes	195 (65.44)	196 (39.52)	37 (37.76)	42 (68.85)	
Stroke location, *n* (%)					0.434
Left side	92 (30.87)	169 (34.07)	31 (31.63)	25 (40.98)	
Right side	109 (36.58)	173 (34.88)	34 (34.69)	14 (22.95)	
Both sides	97 (32.55)	154 (31.05)	33 (33.67)	22 (36.07)	
Diabetes, *n* (%)					0.029
No	118 (39.60)	256 (51.61)	70 (71.43)	20 (32.79)	
Yes	180 (60.40)	240 (48.39)	28 (28.57)	41 (67.21)	
Stroke type, *n* (%)					0.653
Ischaemic	139 (46.64)	240 (48.39)	51 (52.04)	28 (45.90)	
Haemorrhagic	159 (53.36)	256 (51.61)	47 (47.96)	33 (54.10)	
Times since stroke, n (%)					0.481
< 1 month	51 (17.11)	90 (18.15)	19 (19.39)	12 (19.67)	
1–3 months	62 (20.81)	100 (20.16)	14 (14.29)	9 (14.75)	
3–6 months	69 (23.15)	107 (21.57)	20 (20.41)	14 (22.95)	
6–12 months	62 (20.81)	92 (18.55)	21 (21.43)	14 (22.95)	
> 12 months	54 (18.12)	107 (21.57)	24 (24.49)	12 (19.67)	
Tube feeding, n (%)					0.15
No	188 (63.09)	419 (84.48)	77 (78.57)	36 (59.02)	
Yes	110 (36.91)	77 (15.52)	21 (21.43)	25 (40.98)	
Days of hospitalisation	5.69 ± 1.83	5.58 ± 1.78	5.70 ± 1.73	5.15 ± 1.82	0.404
Barthel score	48.06 ± 29.74	51.60 ± 28.30	51.40 ± 24.83	55.18 ± 24.58	0.245
NRS2002 score	4.43 ± 2.82	4.38 ± 2.92	4.80 ± 2.94	3.72 ± 2.74	0.914
BMI (kg/m^2^)	19.94 ± 2.05	21.38 ± 3.06	20.19 ± 2.34	21.42 ± 3.16	0.644
NIHSS score	7.24 ± 5.39	5.84 ± 3.81	7.93 ± 4.71	5.72 ± 3.39	0.588
C-reactive protein (mg/L)	8.21 ± 4.43	6.16 ± 3.89	8.41 ± 4.54	6.64 ± 4.25	0.289
Serum total cholesterol (mg/dL)	197.38 ± 60.60	204.35 ± 58.88	192.45 ± 59.70	205.25 ± 61.07	0.789
Serum albumin (g/dL)	4.49 ± 0.60	4.53 ± 0.57	4.52 ± 0.81	4.66 ± 0.96	0.243

### Results of variables selection

**Fig. 1:**
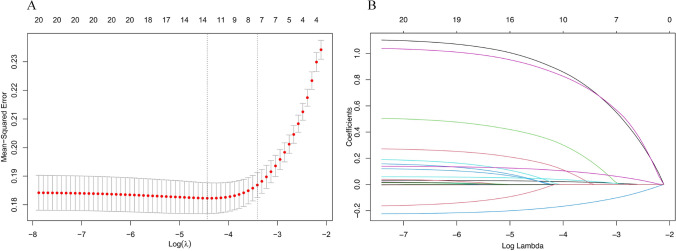
Variable selection using LASSO regression analysis. **A** Cross-validation curve for selecting independent variables. **B** The variation characteristics of the coefficient of variables

The LASSO regression analysis results are presented in Fig. 1. At λ = 0.033, seven variables were selected from the 20 initially analysed: age, limb dysfunction, diabetes, tube feeding, BMI, NIHSS score and C-reactive protein. Multivariate logistic regression analysis (Table 2) confirmed all seven variables as statistically significant predictors (*P* < 0.05). These factors were used to construct the prediction model.

**Table 2:** Multivariate logistic regression analysis of the seven variables

Variable	β	SE	z	Wald χ^2^	OR	95% CI
Age	0.032	0.007	4.318	18.648	1.032	1.017–1.047
Limb dysfunction	1.116	0.173	6.444	41.529	3.053	2.174–4.287
Diabetes	0.522	0.172	3.044	9.263	1.685	1.204–2.359
Tube feeding	1.063	0.198	5.379	28.937	2.894	1.965–4.263
BMI	−0.222	0.036	−6.115	37.393	0.801	0.746–0.860
NIHSS score	0.060	0.020	3.048	9.289	1.062	1.022–1.103
C-reactive protein	0.139	0.021	6.562	43.056	1.149	1.102–1.198

### Model construction and evaluation results

**Fig. 2:**
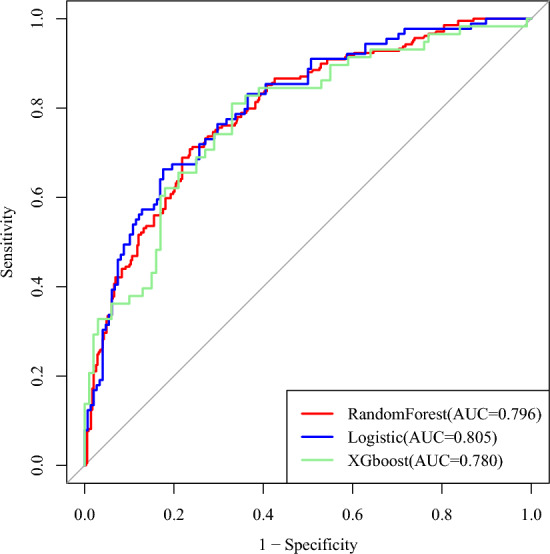
The AUC of the predictive models

**Fig. 3:**
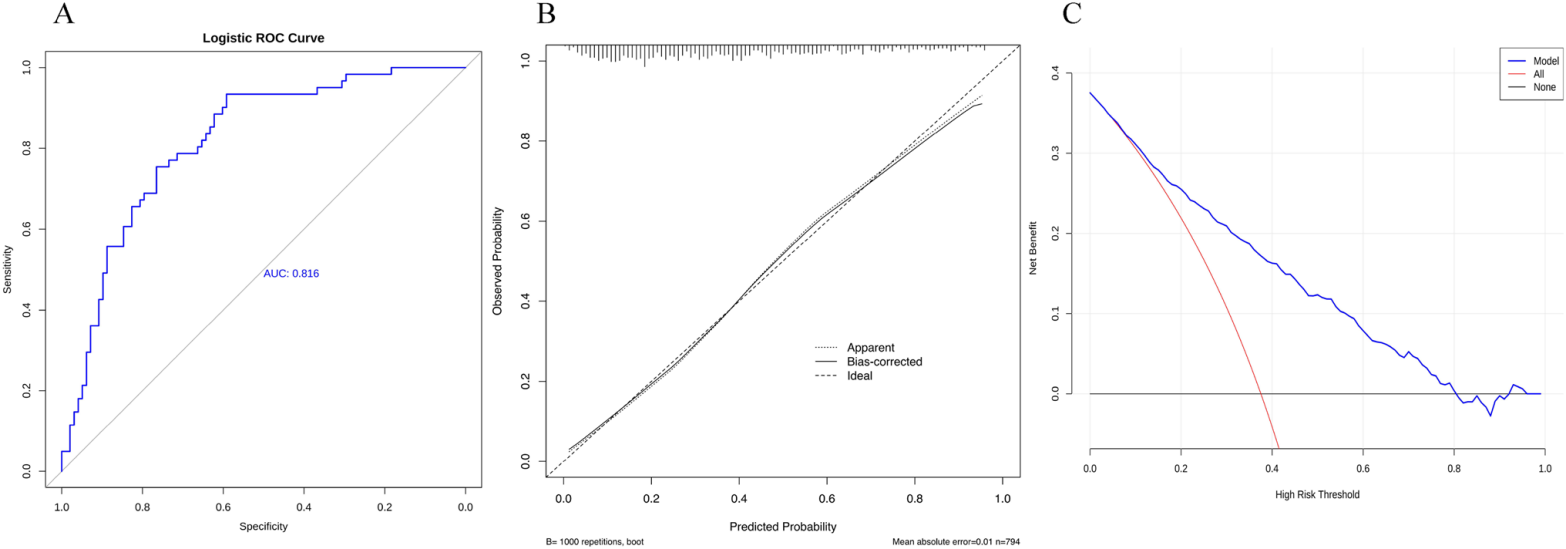
External validation results of the LR model. **A** The ROC curve and AUC value of the model. **B** The calibration curve. **C** Decision Curve Analysis (DCA)

**Fig. 4:**
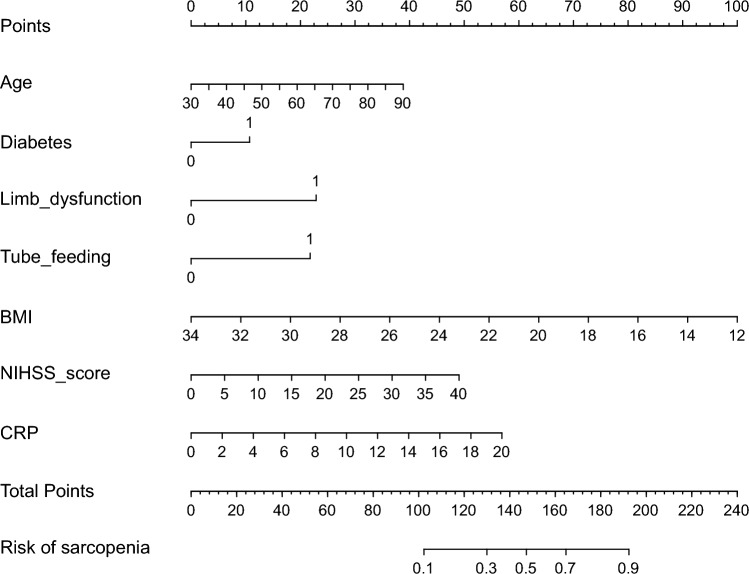
Nomogram for predicting the risk of sarcopenia in patients with stroke

The performance metrics of the LR, RF and XGBoost models are summarised in Table 3. Among the models, the LR model demonstrated the best performance across all metrics. The AUC values were 0.805 for LR, 0.796 for RF and 0.780 for XGBoost (Fig. 2). Based on its superior performance, the LR model was selected for external validation. During external validation, the LR model achieved an AUC of 0.816 (Fig. 3A) and the HL test yielded a P-value of 0.128 (P > 0.05), indicating a good model fit. The calibration curve (Fig. 3B) showed that the bias-corrected line closely aligned with the ideal line, with a mean squared error of 0.01, demonstrating excellent calibration. DCA results (Fig. 3C) indicated that the net benefit of the LR model exceeded the ‘None’ and ‘All’ lines, confirming its strong clinical utility. To enhance clinical applicability, a nomogram was constructed to visually represent the stroke-related sarcopenia risk scoring system (Fig. 4). Additionally, an online risk calculator (https://sarcopenia123.shinyapps.io/shinyen/) was developed to facilitate the practical use of the prediction model in clinical settings.

**Table 3:** Comparison of the performance of the three models

	Accuracy	Precision	Recall	Specificity	F1-score
Logistic regression	0.747	0.764	0.767	0.912	0.762
Random forest	0.738	0.667	0.607	0.818	0.635
XGBoost	0.728	0.632	0.621	0.790	0.626

## Discussion

The prevalence of secondary sarcopenia among patients with stroke is notably high, averaging 42% [4], which exceeds the prevalence reported for other chronic diseases. For comparison, sarcopenia prevalence has been reported at 7–29.3% in patients with diabetes, 15.5% in those with COPD and 32.5% in patients with cancer [12–14]. In this study, sarcopenia prevalence was found to be 37.53% and 38.36% in the training and validation cohorts, respectively, aligning with previous findings [15]. The variability in sarcopenia prevalence across studies is largely attributed to the lack of standardised diagnostic criteria. Studies using the AWGS diagnostic criteria reported prevalence rates of 46.7% in China, 52.8% in Turkey and 32.5% in Japan [3, 15, 16]. In contrast, research using the European Working Group on Sarcopenia in Older People (EWGSOP) criteria in the United States reported a significantly lower prevalence of 13.2% [17]. Even when applying the same diagnostic criteria, prevalence rates can vary across populations. For example, a cross-sectional survey in India using the EWGSOP2 tool found prevalence rates of 26.6–39.10% [18], whereas a Canadian study reported much lower rates of 1.48–6.16% [19]. These findings underscore the importance of establishing standardised, universally applicable diagnostic criteria to reduce discrepancies and enhance the comparability of sarcopenia research across different regions and populations. This study employed the AWGS diagnostic criteria, which are tailored to the Asian population. However, we acknowledge that regional differences in diagnostic standards may limit the model's generalizability and external validity, particularly for Western populations. Future studies should evaluate the model's applicability in non-Asian populations to address these limitations. Despite the variability in diagnostic criteria, research consistently highlights a high prevalence of sarcopenia in patients with stroke, underscoring the need for early detection and preventive measures. Prior studies have identified factors such as hyperlipidemia and atrial fibrillation as potential contributors to sarcopenia risk in stroke survivors [20]. This study identified age, limb dysfunction, diabetes, tube feeding, BMI, NIHSS score and C-reactive protein as independent risk factors for SRS.

Consistent with the definition of sarcopenia and previous research findings, its prevalence increases with age, aligning with the majority of previous studies [21–23]. Ageing is associated with a gradual decline in muscle mass, quality and strength, and this pattern is also observed in patients with stroke. Notably, 85% of patients with stroke experience upper limb dysfunction at the onset of the condition, but only one-third recover some degree of limb function, and even then, the recovery is often incomplete [24]. Nerve damage impairs the central nervous system’s regulation of muscle function. Consequently, when limbs lack sufficient muscle activity and contractions, muscle fibres progressively degrade, resulting in reduced muscle mass and strength. The relationship between the severity of neurological damage, as measured by the NIHSS scale, and the development of sarcopenia in patients with stroke remains unclear. Some studies suggest that sarcopenia may contribute to endothelial dysfunction [25], which has been associated with neurological deterioration [26, 27]. However, the underlying mechanisms linking sarcopenia to neurological decline are not yet fully understood. In this study, the sarcopenia group had a higher average NIHSS score (7.24 ± 5.39 in the training cohort and 7.93 ± 4.71 in the validation cohort) compared to the non-sarcopenia group. This finding supports the hypothesis that higher NIHSS scores are correlated with an increased risk of sarcopenia. The complex relationship between diabetes and sarcopenia has been well established, particularly in patients with type 2 diabetes. This connection is primarily mediated by chronic inflammation, insulin resistance, oxidative stress and neuropathy, all of which contribute to the loss of muscle mass and function [28, 29]. Dysphagia, a common complication in patients with stroke, affects over 50% of individuals [30]. In cases where oral intake is difficult or unsafe, tube feeding becomes essential for providing enteral nutrition. Although this approach meets basic metabolic calorie requirements, it may not adequately fulfil the patient’s nutritional needs, potentially delaying the recovery of swallowing function [31]. Prolonged disuse of oral and pharyngeal muscles can lead to degeneration and atrophy, not only impairing swallowing but also increasing the risk of systemic sarcopenia. After a stroke, patients often enter a hypermetabolic state, which elevates energy requirements for recovery. If nutrition provided via nasogastric tubes fails to meet these heightened needs, weight loss and a reduction in BMI may ensue [32]. Consistent with previous studies, this study found that patients with stroke with lower BMI are at a higher risk of developing sarcopenia [17]. CRP, a non-specific marker of systemic inflammation, has been shown in previous research to be elevated in patients with sarcopenia compared to non-sarcopenic individuals [33]. This chronic inflammation accelerates muscle breakdown, impedes recovery and adversely affects neurological and metabolic functions.

This study utilised LR, RF and XGBoost models to build and validate a sarcopenia risk prediction tool for patients with stroke, incorporating seven variables: age, limb dysfunction, diabetes, tube feeding, BMI, NIHSS score and CRP. Among these models, the LR model demonstrated superior performance, achieving the highest AUC, specificity and sensitivity, indicating its greater accuracy in predicting sarcopenia risk. Consistent with previous studies, the nomogram constructed based on the LR model showed excellent predictive performance, with an AUC value as high as 0.97. Similar research has also identified age and BMI as critical variables in constructing predictive models [34, 35]. However, this study introduced additional variables and optimised the nomogram into an online risk calculator to enhance its clinical utility. The online risk calculator enables clinicians to quickly and accurately assess a patient’s risk of sarcopenia, facilitating the development of personalised treatment plans. For high-risk patients, the calculator highlights the need for targeted interventions. Based on the seven variables incorporated in the model, the tool offers recommendations such as adjusting the frequency and intensity of exercise training, controlling inflammation, managing blood sugar levels, and providing nutritional support. Furthermore, the online platform empowers patients by enabling self-monitoring of their risk levels. Patients can receive tailored advice on regular exercise, dietary modifications and lifestyle adjustments, allowing them to take proactive measures to enhance muscle function and mitigate the progression of sarcopenia. Implementing these interventions can not only reduce the likelihood of adverse outcomes in high-risk patients but also promote rehabilitation and effective health management, ultimately improving their prognosis and quality of life.

Despite its strengths, this study has several limitations. First, the lack of a unified diagnostic standard for sarcopenia—given the differing cutoff values used in Asia and Europe—necessitates caution when generalising these results to other populations. Second, as a single-centre study, while the model demonstrated good clinical utility, its findings require further validation in multi-centre studies with larger sample sizes. Third, the retrospective design may have overlooked relevant variables, such as cognitive impairment and psychological status, which should be considered in future research.

## Conclusion

This study developed and compared three machine learning models to predict sarcopenia risk in patients with stroke. Among these, a nomogram incorporating seven predictive factors—age, limb dysfunction, diabetes, tube feeding, BMI, NIHSS score and CRP—was established as a practical tool for early sarcopenia screening. The nomogram, complemented by an easy-to-use online risk calculator, facilitates the identification of high-risk patients and supports timely interventions. These tools hold the potential to enhance clinical decision-making and improve outcomes for stroke patients at risk of sarcopenia.

## Data Availability

The data that support the findings of this study are available from the corresponding author, upon reasonable request.

## Declarations

### Human and animal right

All procedures followed have been performed in accordance with the ethical standards laid down in the 1964 Declaration of Helsinki and its later amendments.
